# Adoption of an “Open” Envelope Conformation Facilitating CD4 Binding and Structural Remodeling Precedes Coreceptor Switch in R5 SHIV-Infected Macaques

**DOI:** 10.1371/journal.pone.0021350

**Published:** 2011-07-08

**Authors:** Ke Zhuang, Andres Finzi, Silvana Tasca, Madina Shakirzyanova, Heather Knight, Susan Westmoreland, Joseph Sodroski, Cecilia Cheng-Mayer

**Affiliations:** 1 Aaron Diamond AIDS Research Center, New York, New York, United States of America; 2 Division of AIDS, Department of Cancer Immunology and AIDS, Department of Pathology, Dana-Faber Cancer Institute, Harvard Medical School, Boston, Massachusetts, United States of America; 3 Division of Comparative Pathology, New England Primate Research Center, Harvard Medical School, Southborough, Massachusetts, United States of America; The University of Hong Kong, Hong Kong

## Abstract

A change in coreceptor preference from CCR5 to CXCR4 towards the end stage disease in some HIV-1 infected individuals has been well documented, but the reasons and mechanisms for this tropism switch remain elusive. It has been suggested that envelope structural constraints in accommodating amino acid changes required for CXCR4 usage is an obstacle to tropism switch, limiting the rate and pathways available for HIV-1 coreceptor switching. The present study was initiated in two R5 SHIV_SF162P3N_-infected rapid progressor macaques with coreceptor switch to test the hypothesis that an early step in the evolution of tropism switch is the adoption of a less constrained and more “open” envelope conformation for better CD4 usage, allowing greater structural flexibility to accommodate further mutational changes that confer CXCR4 utilization. We show that, prior to the time of coreceptor switch, R5 viruses in both macaques evolved to become increasingly sCD4-sensitive, suggestive of enhanced exposure of the CD4 binding site and an “open” envelope conformation, and this correlated with better gp120 binding to CD4 and with more efficient infection of CD4^low^ cells such as primary macrophages. Moreover, significant changes in neutralization sensitivity to agents and antibodies directed against functional domains of gp120 and gp41 were seen for R5 viruses close to the time of X4 emergence, consistent with global changes in envelope configuration and structural plasticity. These observations in a simian model of R5-to-X4 evolution provide a mechanistic basis for the HIV-1 coreceptor switch.

## Introduction

The human immunodeficiency virus (HIV) enters target cells via interaction of the viral glycoprotein with the cellular receptor CD4 and chemokine coreceptors, either CCR5 (R5 viruses) or CXCR4 (X4 viruses) [1]. Regardless of the route of transmission, R5 viruses account for most of the primary HIV-1 infections [2], [3]. With time, X4 variants arise and coexist with R5 viruses in ∼50% of subtype B infected individuals, and their emergence is associated with accelerated CD4+ T cell loss and disease progression [4]. The determinant of phenotypic change from R5 to X4 maps largely to the V3 loop of the envelope gp120 [5], [6], [7], requiring only a few amino acid substitutions in this region to expand or alter coreceptor preference [8], [9], [10]. Given the minimal requirement for V3 sequence change to confer the ability to use CXCR4, the high levels of virus replication and associated error rate [11], [12], [13], and the selective advantage of expanded target cell population in vivo [14], [15], it is surprising that the switch from R5 to X4 virus does not occur more rapidly and frequently in HIV-1 infected individuals. Although the mechanistic basis and blockade(s) for virus coreceptor switch remain ill-defined, several selective factors such as high viral load and evolutionary rate, CD4+CCR5+ target T cell limitation, and weakening of immune-driven pressures have been proposed as playing important roles [16], [17], [18].

We recently developed a simian model of coreceptor switching, based on infection of rhesus macaques with a pathogenic R5 SHIV isolate, SHIV_SF162P3N_ [19], [20], [21]. The macaques infected intravenously or intrarectally with SHIV_SF1623N_ in which X4 virus evolved and emerged were rapid progressors (RPs), with a clinical course that was characterized by extremely high levels of virus replication and weak or undetectable antiviral antibody and cellular immune responses. Sequence changes in the V3 loop of envelope gp120 were shown to determine the phenotypic change from R5 to X4 in macaques, and this process transitioned through dual-tropic (R5X4) variants capable of using both coreceptors, albeit with reduced efficiency [22]. Interestingly, while X4 appearance was associated with an accelerated drop in peripheral CD4+ T cell count, it followed rather than preceded the onset of precipitous CD4+ T cell loss in infected animals. The newly emerging R5X4 and X4 viruses were highly sensitive to neutralization with soluble CD4 (sCD4), and V3 sequence changes that confer CXCR4 usage are also sufficient to determine increase sCD4 sensitivity of the virus [22]. The conditions (e.g., extremely high levels of virus replication), genotypic requirements (i.e., V3 loop sequence changes) and pattern (e.g., emergence of neutralization sensitive X4 variants following the onset of CD4+ cell loss) for coreceptor switching in SHIV_SF162P3N_-infected macaques overlapped with those reported for HIV-1 infected humans [8], [9], [23], [24], [25], [26], [27], [28], supporting the use of this infection model to study the basis and underlying selection pressures for R5-to-X4 virus evolution in vivo.

In this respect, the findings in HIV-1-infected individuals and in SHIV_SF162P3N_-infected macaques that the emerging R5X4 and X4 variants were highly sensitive to sCD4 neutralization, and that the V3 sequence substitutions that altered coreceptor preference of the virus also determined its sCD4 sensitivity are noteworthy [19], [22], [27], [29], [30]. The former suggests that R5-to-X4 evolution is possible only when neutralization antibody selective pressure is absent or diminished with immune deterioration, while the latter implies that the early steps in the R5-to-X4 evolution process may require the same envelope conformation changes that render the virus sCD4 sensitive. Increased sCD4 sensitivity is indicative of enhanced CD4 binding and accessibility of the CD4 binding site, which is usually masked in the structure of the unliganded envelope glycoprotein of primary HIV-1 isolates in order to avoid the binding of potential neutralization antibodies [31], [32], [33], [34]. Since there is a diminished need to resist neutralizing antibodies in the rapidly progressing macaques, and perhaps in HIV-1 infected individuals towards end stage disease as well, when the immune system collapses, enhanced CD4 binding may be best achieved by adoption of an “open” envelope conformation to expose the CD4 binding site [31], [35]. As envelope structural constraints have been suggested to limit the pathways available for coreceptor switching [13], [36], [37], [38], an “open” envelope configuration can also release or minimize such constraints, allowing for greater flexibility in procuring the conformational transitions needed to confer CXCR4 utilization.

We tested this model for the R5-to-X4 phenotypic switch by assessing the sensitivity to sCD4 and a CCR5 antagonist of viruses pseudotyped with CCR5-using envelope gp160s (Envs) amplified over time from RP macaques with coreceptor switch, with these measurements serving as surrogate markers for CD4 and CCR5 utilization efficiencies, respectively [39], [40], [41], [42], [43], [44]. We also examined binding of soluble gp120 to CD4-Ig, as well as the ability of the R5 pseudoviruses to infect target cells that express low levels of the CD4 receptor. This is because, conceivably, the selection factor for viruses to expose the CD4 binding site and to bind CD4 better is to infect target cells that express low levels of the receptor more efficiently. Accordingly, HIV-1 R5 variants that can infect CD4^low^ cells such as macrophages are frequently detected late in disease [45], [46], [47], [48], and macrophages are the major source of virus in SIV-infected RPs at end-stage disease [49]. Moreover, efficient infection of macrophages in vitro correlates with increased CD4 affinity, the capacity to use low CD4 levels, and with increased sensitivity to sCD4 [43], [50], [51], [52], [53], [54], [55], [56]. Lastly, susceptibility of the R5 pseudoviruses to neutralization with T20 and broadly reactive conformational antibodies was also determined, with broad changes in neutralization sensitivity interpreted as indicative of global rearrangements in glycoprotein structure and greater envelope plasticity [57], [58]. These studies suggest that adaptation of an “open” envelope conformation that binds CD4 more efficiently evolves in persisting R5 viruses, and is an early step in the pathway to the coreceptor switch in rhesus macaques.

## Materials and Methods

### Ethics Statement

This work used blood from SHIV infected macaques housed at the Tulane National Primate Research Center (TNPRC) in accordance with the Animal Welfare Act and Guide for the Care and Use of Laboratory Animals. TNPRC is accredited by the Association and Assessment and Accreditation of Laboratory Animal Care (AAALAC #000594). The OLAW animal welfare assurance number for TNPRC is A4499-01 and the USDA registration number is 72-R-0002. Care was provided by a faculty of 8 veterinarians, and 120 animal care technicians, veterinary technicians and enrichment staff. All procedures were performed on anesthetized animals and post-operative analgesics were administered as needed in accordance with IACUC approval. The Tulane University IACUC and the Division of Veterinary Medicine have established procedures to minimize pain and distress through several means. The use of preemptive and post procedural analgesia is required for procedures that would likely cause more than momentary pain or distress in humans undergoing the same procedure. Any deviation from the administration of analgesics according to this policy requires adequate scientific justification from the investigator and approval by the IACUC. Tulane also has a written endpoint policy to minimize potential pain and distress experienced by animals. If the animal becomes ill and/or meets the criteria for the IACUC approved endpoint policy, it will be euthanized using methods consistent with the recommendations of the American Veterinary Medical Association (AVMA) Panel on Euthanasia.

The Tulane IACUC specifically approved this study. And, in accordance with the recommendations of the Weatherall report “The use of non-human primates in research”, all steps were taken to protect animal welfare and to ameliorate suffering in all work involving non-human primates.

### Cells

293T cells and Hela TZM-bl cells expressing CD4, CCR5 and CXCR4 and containing integrated reporter genes for firefly luciferase and β-galactosidase under control of the HIV-1 LTR [59] were maintained in DMEM supplemented with 10% fetal bovine serum (FCS), 100 U/ml penicillin, 100 µg/ml streptomycin and 2 mM L-glutamine. RC49 and JC53 cells, which are clones of HeLa/CD4/CCR5 cells that express low and high levels of CD4 respectively [60], were maintained in the same media. Human peripheral blood mononuclear cells (PBMCs) were prepared by Ficoll gradient centrifugation, stimulated with phytohemagglutinin (PHA, 3 µg/ml; Sigma, St. Louis, MO) in RPMI medium containing 10% FCS, penicillin, streptomycin, L-glutamine and 20 U/ml interleukin-2 (Norvatis, Emeryville, CA). Monocytes were enriched by centrifugation of PBMCs through a 40% percoll cushion followed by plastic adherence, and cultured in RPMI 1640 medium supplemented with 10% FCS and 5% human AB serum for 5–7 days to allow for differentiation into macrophages [61].

### Plasmid constructs and pseudovirus production

For expression of envelope glycoproteins, full-length gp160 coding sequences were amplified from infected macaque PBMC or plasma RT products with primers SH43 (5′-AAGACAGAATTCATGAGAGTGAAGGGGATCAGGAAG-3′) and SH44 (5′-AGAGAGGGATCCTTATAGCAAAGCCCTTTCAAAGCCCT-3′), subcloned into the pCAGGS vector and sequenced for verification. To generate luciferase reporter viruses capable of only a single round of replication, envelope *trans*-complementation assay was used as previously described [62]. Briefly, Env expression plasmid and the NL4.3LucE-R+ vector were cotransfected with polyethylenimine (PEI, Polyscience, Warrington, PA) into 2.5×10^6^ 293T cells plated in 100 mm plate. Cell culture supernatants were harvested 72 hours later, filtered through 0.45-µm filters, and stored at −70°C in 1-ml aliquots. Pseudoviruses were quantified for p24 Gag content (Beckman Coulter, Fullerton, CA).

### Virus infectivity

For assessment of Env infectivity and entry efficiency, 7×10^3^ TZM-bl cells were seeded in 96-well plates 24 hours before use and infected, in triplicate, with 2 ng p24 Gag equivalent of the indicated pseudotyped viruses. Infected cells were cultured for 72 h at 37°C, at which time the cells were harvested, lysed and processed for luciferase activity according to the manufacturer's instructions (Luciferase Assay System; Promega, Madison, WI). Entry, as quantified by luciferase activity, was measured with an MLX microtiter plate luminometer (Dynex Technologies, Inc., Chantilly, VA). For RC49 and JC53 infections, 7×10^3^ cells were seeded in each well of a 96-well plate on the day prior to infection. Infections were performed in duplicate with 2 ng p24 Gag equivalent of the indicated pseudoviruses, and cells harvested for quantitation of luciferase activity 72 hours later. For infection of primary cells, 10^6^ and 10^5^ cells of human PBMCs and macrophage respectively were infected in duplicate with 5 ng p24 Gag equivalent of the indicated pseudotyped viruses in each well of a 96-well plate. Infected cultures were harvested 72 hours later and processed for luciferase activity. To control for differences in Env entry efficiencies, infectivity for RC49 cells was expressed as a ratio of the infectivity for these cells compared to the infectivity in JC53 cells. Similarly, infectivity in macrophages was normalized to that achieved in peripheral blood mononuclear cells (PBMCs) from the same donor.

### Receptor and coreceptor usage efficiency

For assessment of receptor usage efficiency, 2 ng p24 equivalent of the indicated pseudoviruses in 50 µl were incubated with equal 4-fold serial dilution volumes of the CD4-IgG2 fusion protein (sCD4; PRO 542, Progenics Pharmaceuticals, Tarrytown, NY) for 1 h at 37°C and then added to cells, in duplicate wells, for an additional 2 hours at 37°C. 100 µl of medium was then added to each well and the virus-protein cultures maintained for 72 hours. Control cultures received virus in the absence of sCD4. At the end of the culture period, the cells were lysed and processed for β-galactosidase activity (Galacto-Star System; Applied Biosystems, Bedford, MA). A neutralization curve was generated by plotting the percentage of neutralization vs sCD4 dilution, and 50% inhibitory concentrations (IC_50_) were determined using the Prism 4 software (GraphPad, San Diego, CA). For assessment of coreceptor usage efficiency, 7×10^3^ TZM-bl cells per well of a 96-well plate were inoculated, in duplicate, with 2 ng p24 Gag antigen equivalent of the indicated pseudovirus in the absence or presence of 4-fold dilutions of the CCR5 antagonist PSC-RANTES. The cells were lysed after 72 hours at 37°C, processed for β-galactosidase activity, and IC_50_ determined using the Prism 4 software.

### Soluble gp120 CD4-Ig binding

To examine CD4 binding, gp120 glycoproteins from 293T transfected cells were metabolically radiolabeled for 48 hours with 100 µCi/mL [35S]-methionine/cysteine ([35S] protein labeling mix; Perkin-Elmer, Waltham, Mass) in Dulbecco's modified Eagle's medium lacking methionine and cysteine and supplemented with 5% dialyzed fetal bovine serum. Radiolabeled proteins released in the culture supernatant were incubated with either a mixture of sera from HIV-1 infected individuals or CD4-Ig [a fusion protein in which the N-terminal two domains of CD4 are linked to the Fc component of immunoglobulin G [63]] in the presence of 70 µl of 10% Protein A-Sepharose (American BioSciences Inc, Boulder, CO) for 2 hr at 37°C. The precipitates were analyzed on NuPAGE Novex Bis-Tris polyacrylamide gels (Invitrogen, Carlsbad, CA), followed by autoradiography and quantification with a PhosphorImager (Molecular Dynamics, Sunnyvale, CA).

### Evaluation of spontaneous and sCD4-induced release of gp120

2.5×10^6^ 293T cells seeded in a 100-mm plate were transfected with Env expression plasmid by the polyethylenimine method. Transfected cells were collected 48 h later, washed twice, resuspended in phosphate-buffered saline and divided in half. sCD4 (0.5 µg/ml) was added to one of the two fractions, and both fractions were incubated for 2 h at 37°C. The supernatants and cells were subsequently collected, and the amounts of gp120 in each fraction were quantitated by ELISA according to the manufacturer's instructions (Advanced Biosciences Laboratories, Inc, Kensington, MD). Gp120 release was determined as a percentage of gp120 present in the supernatants compared to the total amount of Envs found in both the supernatants and the cell lysates. Results shown are expressed as the percentage difference in gp120 release in the presence of sCD4 relative to that seen in the absence of sCD4.

### Neutralization assay

Virus neutralization was assessed using TZM-bl cells in 96-well plates. Briefly, equal volumes (50 µl) of pseudoviruses (2 ng p24 Gag equivalent) and 4-fold serial dilutions of IgG1b12, 447-52D and T20 were incubated for 1 h at 37°C and then added to cells, in duplicate wells, for an additional 2 hours at 37°C. 100 µl of medium was then added to each well and the virus-protein cultures maintained for 72 hours. Control cultures received virus in the absence of blocking agent. At the end of the culture period, the cells were lysed and processed for β-galactosidase activity. A neutralization curve was generated by plotting the percentage of neutralization vs agent dilution, and IC_50_ determined using the Prism 4 software.

### Immunophenotyping of SHIV-infected cells

Identification of SHIV-infected macrophages was accomplished with double-label immunohistochemistry performed as previously described with modifications [64], [65]. Briefly, lymph node sections were deparaffinized in xylene and rehydrated through graded ethanol to tris-buffered saline (TBS) plus tween 20. Endogenous peroxidase activity was blocked by incubation in 3% H_2_O_2_ in PBS. Antigen retrieval was accomplished by microwave heating sections at 95°C for 20 minutes in citrate buffer (DAKO, Carpinteria, CA), followed by 20 minute cooling, and Dako protein block for 10 minutes. The blocked sections were incubated with SIVnef antibody (clone KK75, IgG1; 1∶200) overnight at 4°C then reacted with biotinylated secondary antibody (HAM-b, Dako, 1∶200) for 30 minutes. Sections were detected using standard avidin-biotin peroxidase complex technique (ABC Elite, Vector Laboratories, Burlingame, CA) and DAB chromagen (Dako). Sections were blocked again for 10 minutes with protein block (Dako) and incubated with Iba-1 antibody (Wako Chemicals, Richmond, VA, rabbit polyclonal, 019-19741, 1∶1000) for macrophages for 30 minutes at room temperature followed by biotinylated secondary antibody (GAR-b, Dako, 1∶200) for 30 minutes. Sections were detected using standard avidin-biotin alkaline phosphatase complex technique (Vectastain ABC-AP, Vector Laboratories, Burlingame, CA) and Permanent Red (Dako). Slides were counterstained with Mayer's hematoxylin, rinsed in tap water, coated with Clear Mount (Electron Microscopy Science, Hatfield, PA), air-dried overnight, then coverslipped.

#### Statistical Analysis

Differences in susceptibility to sCD4, IgG1b12, 447-52D and T20, as well as infection of CD4^low^ cells and binding of gp120 to CD4-Ig between the acute (w2 for BR24 and w1 for CA28) and the evolving R5 viruses were examined using Mann-Whitney *U* test. P-values<0.05 were considered statistically significant.

## Results

### Increased sCD4 sensitivity of evolving R5 viruses in a RP with coreceptor switch

**Figure 1:**
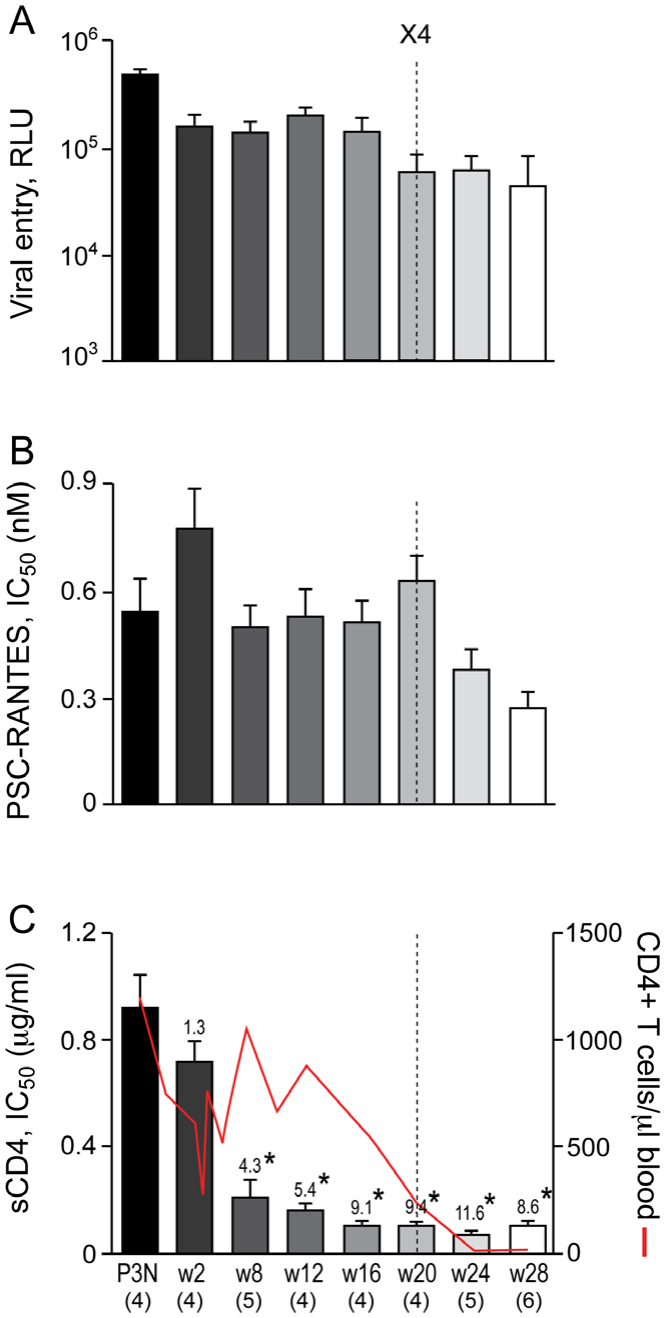
Entry efficiency, PSC-RANTES and sCD4 sensitivity of R5 viruses evolving over time in BR24. Entry of luciferase reporter viruses expressing CCR5-using envelopes into TZM-bl cells expressed as relative light unit (RLU)(A), and susceptibility of the reporter viruses to neutralization with PSC-RANTES (B) and sCD4 (C) were determined. The dashed vertical line indicates time of tropism switch in BR24 (20 wpi), and the numbers in the brackets indicate the number of clones analyzed at each time point. Envelope clones from the SHIV_SF162P3N_ inoculum (P3N) were also included in the characterization for comparison. Absolute CD4+ T-cell count in the animal over the course of infection is shown in (C) for reference, and values above the bars indicate fold increase in sCD4 sensitivity relative to that of the w2 viruses. * P<0.05 (Mann-Whitney *U* test). Data are representative of 2–3 independent experiments (error bars, s.d.).

We first determined if R5 viruses evolved over time to be more sCD4 sensitive in BR24, the initial reported case of coreceptor switching in R5 SHIV_SF162P3N_-infected RP macaques [19]. BR24 sustained high viremia and progressed to disease at 28 week post-infection (wpi) in the absence of seroconversion, with tropism switch documented at 20 wpi. We obtained multiple CCR5-using full-length envelope gp160 (Env) at 2, 8, 12, 16, 20, 24 and 28 wpi and generated single-round replication-competent luciferase reporter viruses for functional characterization. Four randomly selected functional Env clones from the SHIV_SF162P3N_ inoculum were also characterized for comparison. We found no significant difference in the entry efficiency of R5 viruses bearing Envs amplified from macaque BR24 at 2–16 wpi, when measured in CD4^hi^ CCR5^hi^ TZM-bl cells, but R5 viruses present during and following the time of X4 emergence at 20 wpi infected TZM-bl cells less efficiently (2–3 fold reduction in RLU; **Figure 1A**). There was also no significant change in the ability of the evolving R5 viruses to use the CCR5 coreceptor up to the time of switch, as indicated by similar IC_50_ inhibitory dose with the CCR5 inhibitor PSC-RANTES (**Figure 1B**). However, viruses evolving following the time of switch showed a 1.5- to 2-fold increase in susceptibility to PSC-RANTES inhibition, suggesting that they used the CCR5 coreceptor less efficiently. These findings of reduced entry fitness and decreased CCR5 use for R5 viruses that coexist with emerging X4 viruses in BR24 towards end-stage disease at 20, 24 and 28 wpi contrast with reports of increased replication and efficacy of CCR5 usage with disease progression in HIV-1 infected individuals with R5 viruses only [44], [66], [67], [68], [69], [70], [71], [72], but are consistent with results for late R5 viruses from HIV-1 infected individuals with detectable CXCR4-using variants [73], [74], [75].

In contrast, R5 viruses in BR24 evolved prior to the time of coreceptor switch to become increasingly susceptible to inhibition with CD4-IgG2, a tetrameric soluble CD4 (sCD4) construct based on IgG (**Figure 1C**). There was little difference in the concentrations of sCD4 needed to achieve 50% neutralization (IC_50_) of the week 2 (w2) and the inoculating P3N viruses (0.92 and 0.72 µg/ml, respectively), but a statistically significant 4.3-fold increase in sCD4 sensitivity was evident for viruses present six weeks later (w8; IC_50_ of 0.21 µg/ml). sCD4 sensitivity continued to increase significantly for R5 viruses in BR24, with a 5.4-fold increase seen for the w12 viruses (IC_50_ of 0.17 µg/ml), and a 9.1–9.4 fold increase for viruses present at 16 and 20 wpi (IC_50_ of ∼0.1 µg/ml) that is suggestive of increased accessibility of oligomeric gp120 to CD4 prior to and during the time of coreceptor switch. Notably, acquisition of increased sCD4 sensitivity of the R5 viruses took place in the presence of a high CD4+ T cell count (>500 CD4+ T cells per µl blood at 16 wpi), suggesting that paucity of CD4+ target T cells is not the driving force for viruses to expose their CD4 binding site. Increase in sCD4 sensitivity continued for R5 viruses evolving following the time of coreceptor switch in BR24. Compared to the w2 viruses, the w24 and w28 viruses exhibited 11.6- and 8.6-fold increases in sensitivity, respectively.

### The increase in sCD4 sensitivity of early R5 viruses in BR24 correlates with better CD4 binding and with infection of CD4^low^ cells, but this association dissipates near the time of coreceptor switch

**Figure 2:**
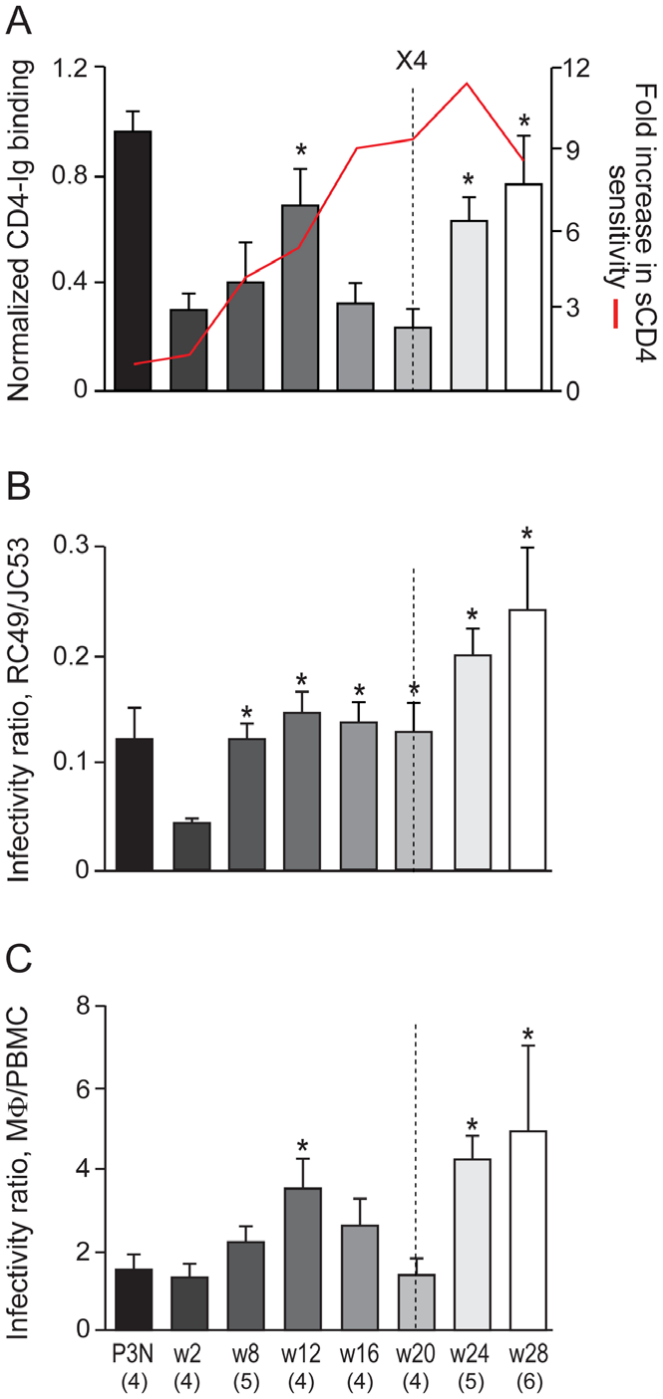
sgp120 CD4-Ig binding and infection of CD4^low^ cells with BR24 viruses. The binding of sgp120 to CD4-Ig together with the fold-increase in sCD4 sensitivity (A), infectivity of HeLa RC49 cells (B) and primary macrophages (mΦ; C) that express low levels of CD4 with pseudotyped viruses bearing CCR5-using Envs amplified over time from BR24 were determined. Properties of four envelope clones in the SHIV_SF162P3N_ inoculum (P3N) were also determined and shown for reference. sgp120 binding to CD4-Ig (A) was normalized to that of sgp120 binding to polyclonal serum from HIV-1 infected individuals. Infectivity in RC49 cells (B) and macrophages (C) that express low levels of CD4 was expressed as a ratio of infectivity in JC53 cells and autologous PBMCs that express high levels of CD4 and CCR5, respectively. The dashed vertical line indicates time of tropism switch. For sgp120 CD4-Ig binding, data are the means and standard deviations from at least two independent experiments. For infection of CD4^low^ cells, data are representative of at least 3 independent experiments (error bars, s.d.). * above the bars indicates normalized CD4-Ig binding and CD4^low^ cell infectivity ratios that are statistically different between the acute (w2) and the evolving R5 viruses.

We next sought to establish, for the evolving R5 viruses in BR24, an association between sCD4 sensitivity, soluble gp120 (sgp120) binding to CD4-Ig, infection of primary macrophages and HeLa RC49 cells, the latter having been used as an indicator of macrophage-tropism and ability to utilize low levels of CD4 for infection [43], [76]. Results showed that the 4.3 and 5.4 fold increase in sCD4 sensitivity of the w8 and w12 viruses as compared to the w2 viruses respectively was accompanied by a 2–3 fold increase in binding of the w8 and w12 sgp120s to CD4-Ig (**Figure 2A**), and with a corresponding fold increase in the ability of the viruses to infect RC49 cells (**Figure 2B**) and primary macrophages (**Figure 2C**). The increase in the ability of the w12 viruses to infect CD4^low^ cells and to bind CD4-Ig is statistically significant. Thus, viruses in BR24 are evolving early to adopt an “open” Env conformation in order to bind CD4 more efficiently for infection of CD4^low^ cells.

**Figure 3:**
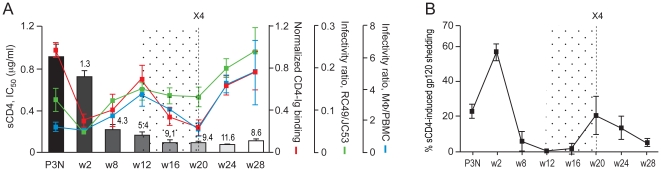
Relationship between sCD4 sensitivity, CD4-Ig binding, infection of CD4^low^ cells and sCD4-induced gp120 release of BR24 viruses. (A) The relationship between sgp120 binding to CD4-Ig, sCD4 sensitivity, infection of RC49 cells and primary macrophages (mΦ) of BR24 dervied viruses is illustrated. Values above the bars indicate fold increase in sCD4 sensitivity of BR24 viruses compared to viruses in the SHIV_SF162P3N_ inoculum (P3N). (B) Extent of sCD4-induced gp120 from surface of 293T cells transiently expressing BR24-derived envelope glycoproteins. Percentage difference in gp120 release in the presence of sCD4 relative to that in the absence of sCD4 is shown. The data are the means and standard deviations of two independent experiments. The vertical dashed line in (A) and (B) indicates the time of coreceptor switching, and the dotted area highlights the time when the relationship between sCD4 sensitivity, sgp120 binding to CD4-Ig and infection of CD4^low^ cells dissipates.

The association between sCD4 sensitivity, sgp120 CD4-Ig binding and infection of CD4^low^ cells was also seen for R5 viruses evolving following the time of coreceptor switch at 20 wpi in BR24. The late w24 and w28 viruses were highly susceptible to sCD4 inhibition (IC_50_ of 0.08 and 0.11 µg/ml, respectively), and bound CD4-Ig and infected CD4^low^ cells with great efficiencies. The notable exceptions were R5 viruses present close to (16 wpi) and at the time of switch (20 wpi). As illustrated in **Figure 3A**, despite an increase in sCD4 sensitivity when compared to the w12 viruses, the w16 and w20 sgp120s exhibited decreased CD4-Ig binding. Moreover, infectivity of the w16 and w20 viruses for CD4^low^ cells was either comparable to that of the w12 viruses (in RC49 cells) or reduced (in primary macrophages).

To understand the disconnect between sCD4 neutralization and CD4 binding of the w16 and w20 viruses, we assessed sCD4-induced gp120 release from the surface of 293T cells transiently expressing their envelope glycoprotein trimers, as this had been shown to be a mechanism by which sCD4 neutralizes HIV-1 infection [77], [78], [79], [80], [81]. Results showed that the extent of sCD4-induced gp120 detachment from the surface of Env-expressing 293T cells was negligible for the w8, w12 and w16 Envs (**Figure 3B**). sCD4-induced gp120 release increased for the w20, w24 and w28 viruses as compared to the w16 virus, but was lower than that of the sCD4-resistant inoculating and w2 viruses. These findings are consistent with previous studies with HIV-1 primary isolates, showing a lack of correlation between sCD4 inhibition and the degree of sCD4-dependent gp120 release [80], [82]. Thus, we conclude that high sCD4 sensitivity of the w16 and w20 R5 viruses in BR24 cannot be explained by increased affinity of the envelope glycoprotein complex for CD4, or by increased sCD4-induced gp120 shedding.

### Global changes in envelope glycoprotein structure of R5 viruses near and at the time of coreceptor switch in macaque BR24

Binding to CD4 induces major conformational changes in the envelope glycoprotein that play key roles in Env-mediated fusion. Among these are exposure of the V3 loop and formation of the coreceptor-binding site on gp120 [83], [84], [85], [86], [87], and of a triple-stranded coiled coil activated fusion intermediate structure composed of the N-terminal heptad repeat (HR1) region of gp41 [88], [89], [90], [91], [92], [93]. Structural alterations in the gp120 CD4 binding site or in the V3 domain for BR24 viruses present near or at the time of coreceptor switch, therefore, could have affected their sCD4 susceptibility. Furthermore, recent studies showed that induction of an activated state in the HIV-1 Env that rapidly decays into functionally inactive forms could also mediate sCD4 sensitivity [94]. Accordingly, we assessed neutralizing antibody and T20 sensitivity of the w16 and w20 viruses to probe the conformational state of their envelope glycoproteins. The antibodies used were the broadly neutralizing antibody IgG1b12 directed against the CD4 binding site, and the anti-V3 loop MAb 447-52D [95], [96]. The fusion inhibitor T20 (also known as Fuzeon or enfuvirtide) binds to the hydrophobic groove on the surface of the coiled coil formed by HR1 [88], [97], and sensitivity to T20 has been shown to be modulated by gp120 interactions with the coreceptor as well as the half-life of the HR-1 groove [94], [98], [99], [100].

**Figure 4:**
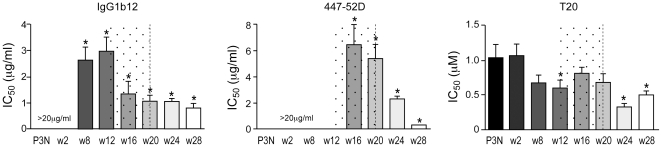
Changes in neutralization sensitivity of R5 viruses evolving over time in macaque BR24. Susceptibility of BR24 R5 pseudoviruses to neutralization with b12, 447-52D and T20 was determined, with sensitivity of variants from the inoculating virus SHIV_SF162P3N_ (P3N) shown for reference. The vertical dashed line indicates the time of coreceptor switching, and the dotted area designates the period of marked envelope conformational changes. Data are representative of at least two independent experiments (error bars, s.d.). * above the bars indicate IC_50_ values that are statistically different between the acute (w2) and the evolving R5 viruses.

Statistically significant changes in virus sensitivity to b12 and 447-52D were evident beginning at 8 and 16 wpi, respectively (**Figure 4**). Notably, there was an increase in b12 sensitivity that may be indicative of structural changes in CD4 binding site conformation or accessibility. 50% inhibition of the w16 and w20 viruses was achieved with ∼1 µg/ml of the antibody compared to 3 and >20 µg/ml needed for the earlier (w8 and w12) and acute (w2) viruses, respectively. The w16 and w20 viruses were also more sensitive to 447-52D neutralization as compared to the earlier R5 viruses (IC_50_ of 5–6 µg/ml compared to >20 µg/ml, respectively), with continued increase in sensitivity for viruses following the time of switch, perhaps suggestive of increased exposure of the V3 loop. Furthermore, the w16 viruses were slightly more resistant to T20 neutralization (IC_50_ of 0.8 µg/ml) compared to the early (w8 and w12; IC_50_, ∼0.6 µg/ml) as well as the late (w24 and w28; IC_50_, 0.3–0.5 µg/ml) viruses; one possible explanation is that exposure of the gp41 HR1 groove on envelope glycoproteins of w16 viruses decays faster. Together, these findings are in support of significant changes in structure or accessibility of the CD4 and the V3 loop, and easier induction of a metastable activated state of the envelope glycoprotein which could account for the increase in sCD4 sensitivity of the w16 and w20 R5 viruses.

### Similar early events for tropism switch in another SHIV_SF162P3N_ infected RP macaque

**Figure 5:**
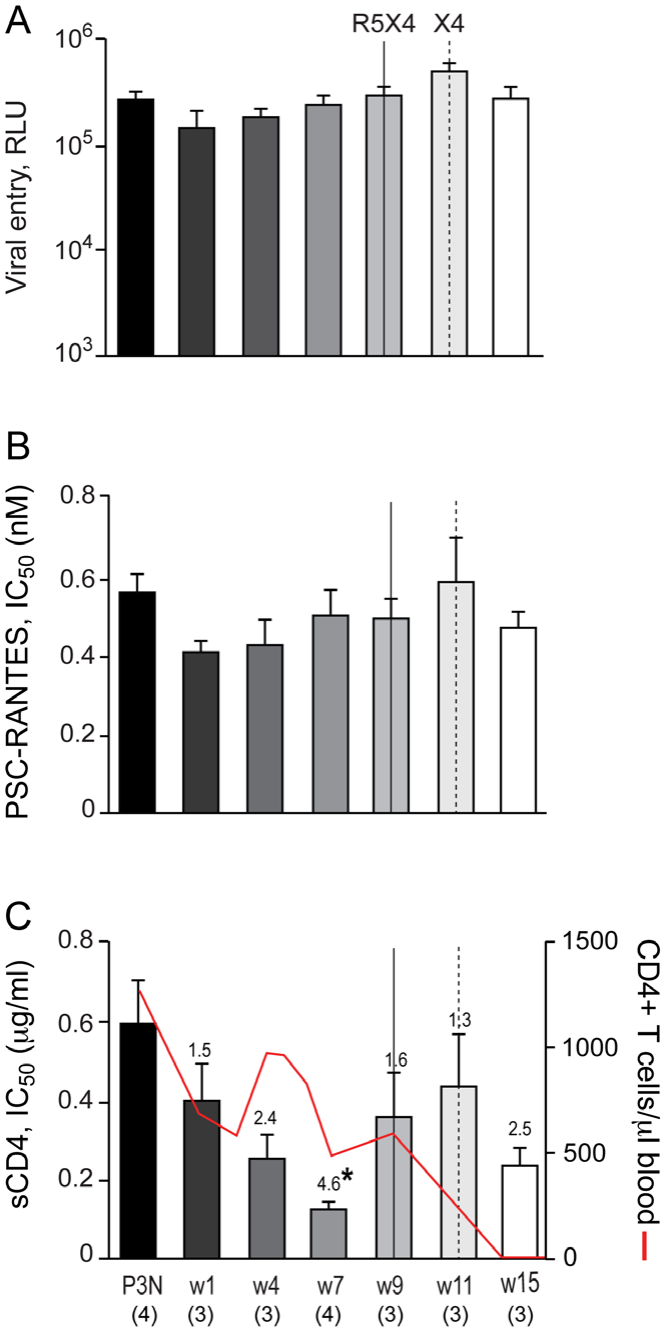
Entry efficiency, PSC-RANTES and sCD4 sensitivity of R5 viruses evolving over time in CA28. Entry of luciferase reporter viruses expressing CCR5-using envelopes into TZM-bl cells (A), and susceptibility of the reporter viruses to neutralization with PSC-RANTES (B) and sCD4 (C) were determined. The solid and dashed vertical lines indicate the two switch events in CA28 leading to the emergence of distinct dual-tropic and X4 viruses, respectively. The numbers in the brackets denote the number of envelope clones analyzed at each time point. Absolute CD4+ T-cell count in the animal over the course of infection is shown in (C), and values above the bars indicate fold increase in sCD4 sensitivity of CA28 viruses compared to viruses in the SHIV_SF162P3N_ inoculum (P3N). *P<0.05 (Mann-Whitney *U* test). Data are representative of at least three independent experiments (error bars, s.d.).

To corroborate the above findings, we investigated if the early events of envelope evolution prior to the time of switch in BR24 are recapitulated in CA28, another RP macaque with coreceptor switch. Peak and set-point viremia were one-log higher in CA28 than in BR24, and the animal progressed to AIDS at 15 wpi, with transient seroconversion at 7 wpi [20]. We previously documented X4 emergence in CA28 at 11 wpi, but more recent studies revealed the presence of another R5-to-X4 evolutionary pathway that led to the emergence of a distinct dual-tropic virus at 9 wpi [101]. We amplified CCR5-using Envs from CA28 at w1, 4, 7, 9, 11 and 15 wpi and found them to mediate comparable entry into TZM-bl cells (**Figure 5A**). There was no notable difference in PSC-RANTES sensitivity of the evolving R5 viruses (<2-fold, **Figure 5B**), but consistent with findings in BR24, R5 viruses in CA28 prior to the time of tropism switch were more sensitive to sCD4 neutralization (**Figure 5C**). Compared to the w1 viruses, the w7 viruses from CA28 were significantly more sensitive to sCD4 neutralization. And, as was observed in BR24, the increase in sCD4 sensitivity was acquired in the presence of high CD4+ T cell numbers (∼500 CD4+ T cells per µl blood at 7 wpi). sCD4 sensitivity however decreased for viruses during the time of switch in CA28 (w9 and w11 viruses). The transient development of anti-SHIV antibody at 7 wpi in this animal could be a contributing factor.

**Figure 6:**
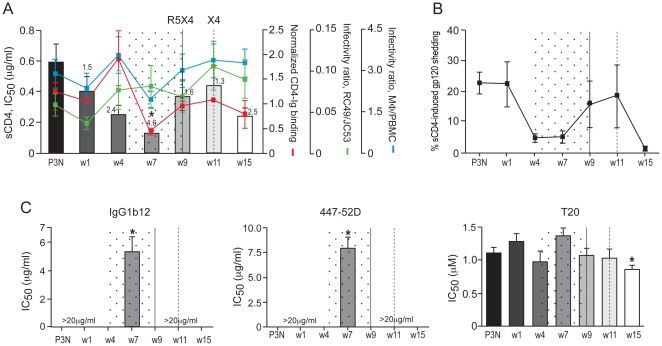
Structure and function of R5 viruses evolving over time in macaque CA28. The relationship between sCD4 sensitivity, binding of sgp120 to CD4-Ig, infectivity of HeLa RC49 cells and primary macrophages (mΦ)(A), the extent of sCD4-induced gp120 release (B), and neutralization susceptibility (C) of pseudoviruses bearing CCR5-using Envs amplified over time from CA28 is shown. The solid and dashed vertical lines indicate time of the two switch events in CA28, and the dotted area marks the time when correlation between sCD4 sensitivity, sgp120 binding to CD4-Ig and infectivity of CD4^low^ cells dissipates (A), and period of notable envelope conformational change (B and C). Data are representative of at least two independent experiments (error bars, s.d.). * above bars indicate differences in sCD4 sensitivity, CD4-Ig binding and susceptibility to agents and antibodies between the acute (w2) and the evolving R5 viruses that are statistically significant.

The 2.4-fold increase in sCD4 sensitivity of the w4 viruses correlated with a corresponding fold increase in sgp120 binding to CD4-Ig and with enhanced infection of RC49 cells and primary macrophages (**Figure 6A**), but this association dissipated at w7, two weeks prior to the first switch event in this animal. Importantly, and consistent with what was observed for BR24 w16 viruses, the dissociation between sCD4 neutralization and CD4 binding of the CA28 w7 viruses cannot be explained by greater sCD4-induced gp120 release (**Figure 6B**), but by antigenic change in the receptor binding site and the V3 loop (**Figure 6C**). The extent of sCD4-induced gp120 release was comparable for the w4 and w7 viruses, but 50% neutralization of the w7 viruses was achieved with ∼5 µg/ml of the anti-CD4BS antibody b12 and ∼8 µg/ml of the anti-V3 mAb 447-52D as compared to >20 µg/ml for the w4 and viruses present at the other time points examined. The increase in sensitivity to b12 and 447-52D neutralization of the w7 viruses is statistically significant. Furthermore, the w7 viruses showed a modest increase in T20 resistance (IC_50_ of 1.4 µg/ml in comparison to ∼1 µg/ml for the other viruses). Collectively, the similarities in increase sCD4 sensitivity that is associated with better CD4 binding of the early R5 viruses, and changes in envelope configuration for R5 viruses close to the time of switch in BR24 and CA28 support similar mechanism(s) and selective pressures for change in coreceptor preference in the two RP macaques.

### Macrophages are the predominant virus-producing cells at end-stage disease in macaques BR24 and CA28

**Figure 7:**
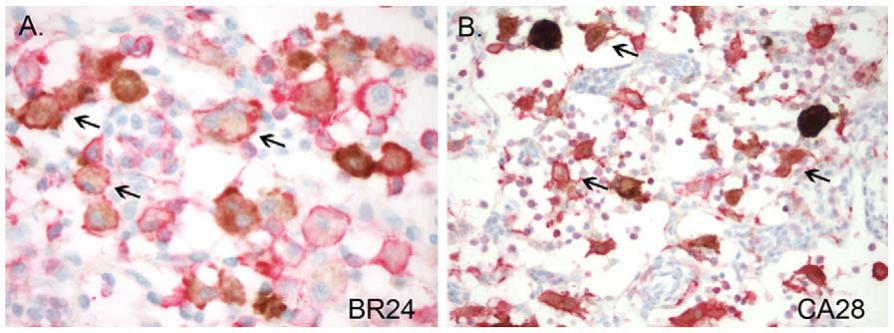
SHIV-infected macrophages identified with double-label SIVnef and Iba-1 immunohistochemistry. Tissue macrophages are the primary SHIV infected cells at end stage disease in BR24 (A) and CA28 (B). Double-labeled immunohistochemical staining for SIVnef (brown) and the macrophage marker lba-1 (red) was performed. Arrows mark representative double-positive cells.

To determine if viruses are evolving in BR24 and CA28 for infection of macrophages in vivo, double labeled immunohistochemical staining for SIV nef (brown) and the macrophage marker lba-1 (red) was used to identify SHIV-expressing cells in the mesenteric lymph node at time of euthanasia. Based on coexpression of lba-1, the majority of SHIV–expressing cells in the lymph node of BR24 and CA28 were found to be macrophages (**Figure 7**). Thus, similar to findings in SIV-infected RPs at end-stage disease [49], macrophage infection is responsible for sustaining virus replication in the two R5 SHIV_SF162P3N_-infected RP macaques at very late stages of disease.

## Discussion

A change in coreceptor preference from CCR5 to CXCR4 late in infection has been well documented in some HIV-1 infected individuals since the early days of the AIDS epidemics, but the reasons and mechanisms for this tropism switch remain elusive. Because X4 emergence is strongly associated with rapid CD4+ T–cell loss and disease progression, and concerns that the introduction of CCR5 entry inhibitors as anti-HIV therapeutics could facilitate X4 emergence and exacerbate disease, there is an increasing need to improve our understanding of the selection pressures which favor CCR5-to-CXCR4 switch. Using a simian model of HIV-1 coreceptor switch, we tested in this study the hypothesis that an early selective force in the evolutionary pathway of tropism switch is the need for viruses to increase the efficiency of CD4 binding for infection of CD4^low^-expressing cells such as tissue macrophages. The adoption of a less constrained and more “open” envelope conformation that exposes the CD4 binding site for enhanced CD4 binding, in turn, releases or reduces envelope structural constraints that have been suggested to limit the pathways available for change in coreceptor preference. We show that R5 viruses evolved early in two rapid progressor macaques to become sCD4-sensitive, and this correlated with better gp120 binding to CD4 and with efficient infection of CD4^low^ cells such as primary macrophages and the HeLa RC49 cells. Furthermore, significant changes in neutralization sensitivity to agents and antibodies directed against functional domains of both gp120 and gp41, including the V3 loop that is important for coreceptor binding were seen for R5 viruses present close to the time of X4 emergence in these rapid progressing macaques, consistent with global changes in envelope conformation and structural plasticity that facilitate the remodeling needed to expand or switch to CXCR4 usage. These observations in two R5 SHIV_SF162P3N_-infected macaques therefore support our proposed mechanistic model for coreceptor switching.

Several mechanisms can explain sCD4 sensitivity of HIV/SIV. For the early R5 viruses in macaques BR24 (w8, w12) and CA28 (w4), we showed that increase sCD4 sensitivity correlated with better CD4-Ig binding (**Figures 3A** **and** **6A**), consistent with exposure of the CD4 binding site and adoption of an “open” envelope conformation. For R5 viruses close to the time of switch (w16 for BR24 and w7 for CA28), however, changes in the CD4 binding site and/or alteration in the conformational changes induced by CD4 binding appeared to be the underlying basis (**Figures 4** **and** **6C**). Interestingly, we observed, in both macaques, that sCD4-induced gp120 shedding decreased for Envs evolving prior to the time of switch (**Figures 3B** **and** **6B**), suggesting that a tighter interaction between the gp120 and gp41 may be necessary during the process of envelope remodeling to acquire CXCR4 use. Alternatively, it has been proposed that an increased number of virion-associated Env complexes available for receptor interaction might facilitate infection of CD4^low^ cells [54], [102], [103]. Thus, it is conceivable that a more stable gp120-gp41 interaction, in particular for BR24 w8 and w12 and CA28 w4 Envs, increases gp120 retention by Env complexes for infection of CD4^low^ cells. Genetic studies to determine if virion-gp120 retention and infection of CD4^low^ cells of these early viruses in BR24 and CA28 are linked will be required to examine this latter possibility.

We show that acquisition of increased sCD4 sensitivity occurred in the presence of high amounts of CD4+ T cells, implying that paucity of CD4+ target T cells is not the driving force for viruses to expose their CD4 binding site and to increase CD4 binding. Moreover, we recently reported that viruses did not evolve early to become sCD4 sensitive in macaques that were depleted of B cells to abrogate or diminish antiviral antibody responses prior to infection with SHIV_SF162P3N_, implying that the reduced antibody-driven pressure in the RPs was also not sufficient to select for viruses with an “open” Env conformation [104]. Rather, the tight association between CD4 binding and infection of CD4^low^ cells of the evolving R5 viruses in both BR24 and CA28, and the finding that primary macrophages are the principle virus-producing cells at end-stage disease in these two macaques with coreceptor switch suggest that adoption of an “open” Env is in response to the need to use low levels of CD4 receptor more efficiently. However, increased sCD4 sensitivity and CD4 binding were seen as early as 4–8 wpi, a time when CD4+ T cells and not tissue macrophages are the preferred targets of HIV/SIV infection [105], [106], [107]. This then raises the intriguing possibility that a selective pressure for altered CD4 affinity of the early R5 viruses in BR24 and CA28 could be decreasing CD4 expression levels on target T cells. Although direct evidence in support is lacking, infectivity of HIV-1 primary isolates in vitro is strongly dependent on the level of CD4 expression [40], [108], [109]. Moreover, transmitted and founder viruses in acute HIV-1 infection have been reported to replicate poorly in monocyte-derived macrophages [3], [110] and to require high receptor levels for entry [76]. Our finding that the ability of the acute viruses (w2 for BR24 and w1 for CA28) to bind CD4 and to infect CD4^low^ cells in both macaques is decreased is consistent with these reports in human, and suggests that CD4+ T cells expressing high amounts of the receptor may be the earliest and preferred targets of virus infection and depletion in vivo, leaving only cells with lower CD4 levels available during the post-acute phase of infection. Nevertheless, CD4 and CCR5 concentration requirements for R5 HIV-1 infections in vitro have been shown to be interdependent, with viruses being highly dependent on the CD4 concentrations or strength of the initial virus-CD4 bond when cell surface CCR5 density is low [60]. Thus, it is possible that the selection factor for better CD4 usage we observed in the RP macaques following acute R5 SHIV_SF162P3N_ infection could be due to initially low CCR5 and not CD4 expressions on T lymphocytes. Studies to monitor variations in CD4 and CCR5 cell surface densities on target T cells during the course of SHIV_SF162P3N_ infection and to examine their relationship to macrophage infection and tropism switch in RP macaques will be needed to more clearly address the selection factors for viruses to evolve early to use low levels of the CD4 receptor more efficiently.

Because most HIV-1-infected individuals have developed neutralizing antibodies, less constrained and “open” envelopes are selected against and not commonly found. This then raises the question as to what extent the observed changes associated with the coreceptor switch in rapid progressor macaques that did not develop or maintain a strong antiviral antibody response reflect what occurs in humans. In this regard, it is noteworthy that X4 dominance is seen only towards end-stage disease in HIV-1 infected individuals, when the immune system is impaired [25], [28], [111], [112]. And, although rare, rapid progressor status has been documented in HIV-1 infected individuals [73], [113], [114], [115], [116], [117], [118], [119], with phenotypic switch reported in cases where this was examined [73], [119]. Moreover, emergence of sCD4 neutralization-sensitive X4 viruses in the presence of neutralizing antibodies has been reported [27], suggesting that X4 virus evolution is in anatomical compartments with lower antibody pressure than in the plasma, and/or that these viruses spread via cell-cell, a mode of transmission that is less susceptible to antibody neutralization. Indeed, we have shown that peripheral lymph nodes that are enriched in target cells for X4 viruses are the preferred sites of their evolution and amplification [21], [22], and the syncytium-inducing/fusion capacity of X4 viruses has been well documented [120]. Thus, the observations made in the SHIV-infected macaques studied here are likely to represent an important step toward our understanding of HIV-1 coreceptor switch in humans.

In summary, our findings provide evidence that adoption of an “open” Env by R5 viruses in response to the selection pressure for better CD4 usage and infection of CD4^low^ cells represents an early step in the chain of events leading to R5-to-X4 evolution, allowing other selection factors such as virus replication-associated mutational events that are required for tropism switch, but which usually come with costs to viral fitness because of structural constraints, to be manifested. Studies of coreceptor switch in RPs are useful for they allow examination of the process of R5 envelope evolution required for a generalized switch uncomplicated by the selection pressure of antiviral antibody responses. Although our studies were limited with respect to the number of animals, the similarity of the evolutionary pattern in structure and function of R5 envelope variants seen in the two outbred RP macaques that differed in the kinetics and levels of virus replication prior to the time of coreceptor switch support a shared mechanism and selective pressure(s) for the change in coreceptor preference. Further research will be required to determine if acquisition of an “open” Env conformation to increase CD4 affinity is a property unique to the early R5 viruses in R5-SHIV_SF162P3N_-infected RPs with coreceptor switch, and how broadly our findings in the SHIV-rhesus model relate to HIV infection of humans. Additionally, it will be of interest to examine coreceptor switching in SHIV_SF162P3N_-infected macaques that have developed a neutralizing antibody response, to discern the impact of humoral immune selection forces on the tempo and molecular pathways available for tropism switch.
